# ﻿Morphological, molecular, and life cycle study of a new species of *Oligogonotylus* Watson, 1976 (Digenea, Cryptogonimidae) from Colombia

**DOI:** 10.3897/zookeys.1115.75538

**Published:** 2022-08-01

**Authors:** Verónica Vélez-Sampedro, Mónica Uruburu, Carolina Lenis

**Affiliations:** 1 Programa de Estudio y Control de Enfermedades Tropicales (PECET), Facultad de Medicina, Universidad de Antioquia, Calle 62 No. 52-59, CP 050010, Medellín, Antioquia, Colombia Universidad de Antioquia Medellín Colombia; 2 Departamento de Ciencias Biológicas, Universidad EAFIT, Carrera 49 No. 7 sur 50, Medellín, Antioquia, Colombia Universidad EAFIT Medellín Colombia

**Keywords:** Blue mojarra, Cauca molly, *Oligogonotylus*, South America, taxonomy, Trematoda

## Abstract

The present study describes *Oligogonotylusandinus***sp. nov.** and its life cycle from a rural fish farm in Sopetrán, Antioquia, Colombia. The endemic species of snail *Aroapyrguscolombiensis* and the fishes *Poeciliacaucana* and *Andinoacaralatifrons* are identified as the first intermediate host, the second intermediate host and the definitive host, respectively. The new species was defined through an integrative approach, combining the traditional morphology of its developmental stages with molecular analyses of the markers ITS2 from ribosomal DNA and COI from mitochondrial DNA. This new species can be distinguished from its congeners by genetic divergence, the position of the vitelline fields, and the number of gonotyls. This work represents the first report of a species of this genus in South America.

## ﻿Introduction

The genus *Oligogonotylus* Watson, 1976 (Digenea: Cryptogonimidae) includes two species, both occurring in Middle America (Choudhury et al. 2016, 2017). These infect the gastrointestinal tract of freshwater cichlid and characid fishes and have a distinctive morphological characteristic of a longitudinal row of gonotyls; these small sucker-like structures are present in a number of flukes in this family. The type species, *Oligogonotylusmanteri* Watson, 1976, is distributed between southeastern Mexico and Costa Rica (Watson 1976; Pérez-Ponce de León et al. 2007; Sandlund et al. 2010). A second species, *O.mayae* Razo-Mendivil, Rosas-Valdez & Pérez-Ponce de León, 2008, is restricted to the Ría Celestun, Estero Progreso at Corchito, and the Ría Lagartos, Yucatán, Mexico (Razo-Mendivil et al. 2008). Both species utilize three hosts in their life cycle; the operculate micromollusk *Pyrgophoruscoronatus* Pfeiffer, 1840 (Cochliopidae) as the first intermediate host (Ditrich et al. 1997; Jiménez-García and Vidal-Martínez 2005; May-Tec et al. 2020), several species of fishes as second intermediate hosts, and the native cichlid fish *Mayaherosurophthalmus* (Günther, 1862) as intermediate and the main definitive host among other 11 species of cichlids (Scholz et al. 1994, 1995; Pérez-Ponce de León et al. 2007; Sosa-Medina et al. 2015; May-Tec et al. 2020). In the present study, we describe a new species of *Oligogonotylus* and its life cycle from a rural fish farm in Colombia using an integrative approach combining ecological, molecular, and morphological data.

## ﻿Methods

### ﻿Sampling

**Figure 1.**
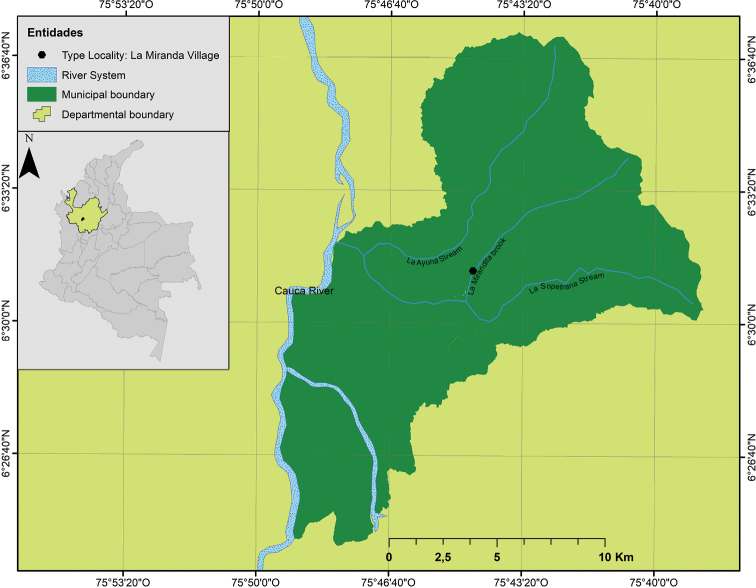
Type locality of the new species of *Oligogonotylus* from La Miranda village, Sopetrán, Antioquia, Colombia. Map made in ArcGIS 18.1.

We conducted fieldwork in La Miranda village, municipality of Sopetrán, Antioquia, Colombia (6°30'42"N, 75°45'22"W; Fig. 1) in March 2019 and February 2020. The sampling was performed in two fishponds supplied by La Mirandita brook in the Middle Cauca river basin. The ponds are approximately 5 m deep, 14 m long, 13 m wide for the first and 15 m wide for the second, with a total volume of 910 m^3^ and 1050 m^3^, respectively. Snails *Aroapyrguscolombiensis* Malek & Little, 1971 (*n* = 451), and fishes of the species *Andinoacaralatifrons* (Steindachner, 1878) (*n* = 22), *Colossomamacropomum* (Cuvier, 1816) (*n* = 6), *Oreochromisaureus* (Steindachner, 1864) (*n* = 6), *O.mossambicus* (Peters, 1852) (*n* = 9), and *Poeciliacaucana* (Steindachner, 1880) (*n* = 105) were collected from the ponds. All animals were transported live to the Laboratory of Helminthology, Programa de Estudio y Control de Enfermedades Tropicales, Facultad de Medicina, Universidad de Antioquia (Medellín, Colombia) and were placed in aquaria prepared with dechlorinated water, artificial aeration, an approximate temperature of 22–24 °C and a controlled photoperiod of 12 h of light and 12 h of darkness.

### ﻿Collection of the cercariae, metacercariae and adults

The search for the parasite specimens was based on standard techniques, cercarial emission in snails (Vergara and Velásquez 2009; Anucherngchai et al. 2016) and organ dissection of fishes to search for parasite infections under a stereo-dissecting microscope. To induce the cercarial emission, the snails were separated and examined individually under a microscope in 1.5 mL Eppendorf tubes with 0.1 mL of distilled water and stimulated under a cold light source for 4 h. To determine the prevalence of infection, non-emitting snails were analysed in triplicate for 21 d. The collected fishes were sacrificed with an overdose of isoeugenol according to Erikson (2011). The muscle of *P.caucana* was examined for the presence of metacercariae, and the digestive tract of *A.latifrons*, *C.macropomum*, *O.aureus*, *O.mossambicus*, and *P.caucana* were examined for the presence of juvenile and adult parasites. The trematodes were rinsed in saline solution, counted, and preserved in 1% glutaraldehyde (cercariae), alcohol-formalin-acetic acid (AFA), and 70% ethanol for morphological identification, or fixed directly in 96% molecular-grade ethanol. The ecological parameters of infections (prevalence, mean intensity and abundance of infection, and intensity range) were calculated according to Bush et al. (1997).

### ﻿Morphological analyses

Cercariae were studied as temporary mounts stained with methylene blue. Metacercariae and adults were dehydrated in an alcohol series, stained with Meyer’s carmine or Borax carmine, cleared in methyl salicylate, and mounted on permanent slides in Canada balsam. A Nikon Alphaphot YS-2 with a Nikon 1.25× drawing tube was used to illustrate the parasites. A Leica DM500 (ICC50 W) microscope was used to analyse and photograph the stained specimens. Morphometric data are given as the mean and range in micrometers (µm). Line drawings and photographic images were prepared using Inkscape 0.92. Type specimens were deposited in the Colección Colombiana de Helmintos, CCH.116, Universidad de Antioquia, Medellín, Colombia.

### ﻿DNA extraction, PCR, and sequencing

The DNA was extracted from individual specimens following the protocol of the genELUTE Mammalian genomic DNA miniprep kit. For barcoding, the nuclear ribosomal second internal transcribed spacer region (ITS2) was amplified using the primer pair: ITS-F 5'-CGGTGGATCACTCGGCTCGT-3' and ITSR 5'-CCTGGTTAGTTTCTTTTCCTCCGC-3' (Iwagami et al. 2000), and the partial cytochrome c oxidase subunit 1 mitochondrial gene (COI) was amplified using the primer pair: JB3 5'-TTTTTTGGGCATCCTGAGGTTTAT-3', and JB4 5'-TAAAGAAAGAACATAATGAAAATG-3' (Bowles et al. 1993). All amplification reactions were as follows: 5 min at 95 °C, followed by 35 cycles each of denaturing (94 °C, 30 s), annealing (50 °C, 45 s), and extension (72 °C, 1 min) for ITS2; annealing (53 °C, 45 s), and extension (72 °C, 1 min) for COI, and 5 min at 95 °C, followed by 35 cycles each for denaturing (94 °C, 30 s). The final extension was done at 72 °C for 5 min. PCR products were purified and sequenced by the Sanger method at Macrogen Inc., Seoul, Korea. The accession numbers in GenBank are given for each barcoded specimen.

### ﻿Phylogenetic tree construction

The ITS2 and partial COI sequences of individual *Oligogonotylus* (ITS2: *n* = 4 adults, *n* = 1 metacercaria, *n* = 1 cercaria; COI: *n* = 6 adults) were edited and assembled in GENEIOUS 2022.0.1 (http://www.geneious.com; Kearse et al. 2012). All sequences were aligned with MUSCLE (Edgar 2004) including sequences of ITS2 and COI of other *Oligogonotylus* species and representative species of other genera of opisthorchids available in GenBank. The best-fitted nucleotide substitution models based on the Bayesian information criterion were estimated in JMODELTEST (Darriba et al. 2012). The genetic divergence (*p*-value) was calculated for each data set using MEGA X (Kumar et al. 2018) and the resulting alignments were analysed using IQ-TREE 2.1.3 (Nguyen et al. 2015) where all phylogenetic trees were constructed using the maximum-likelihood method. The ITS2 phylogenetic tree was based on the symmetrical model with gamma distribution (SYM+G); the COI phylogenetic tree was based on the general time-reversible model with gamma distribution (GTR+G); and COI–ITS2 concatenated tree was based on the general time-reversible model with proportion of invariable sites and gamma distribution (GTR+I+G). Downloaded sequences of some species of the same family (Cryptogonimidae) and another close family (Heterophyidae) were used in all cases as outgroup taxa to ensure the phylogenetic position of the new species. Trees were drawn to scale, and branch-support values were obtained with the ultrafast bootstrap approximation (UFBoot) (Hoang et al. 2018) implemented in IQ-TREE 2.1.3 (Nguyen et al. 2015) based on 1000 bootstrap replicates.

## ﻿Results

**Figure 2.**
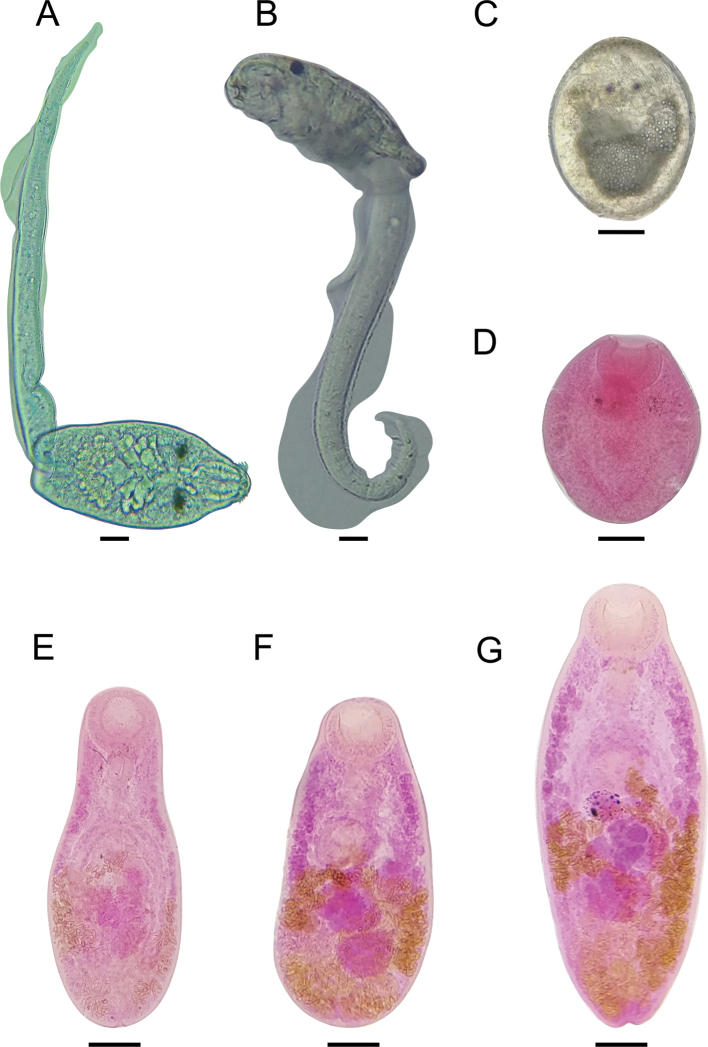
Micrographs of different stages of *Oligogonotylusandinus* sp. nov. **A, B** cercariae emerged from *Aroapyrguscolombiensis*, as temporary mounts **C** encysted metacercaria from *Poeciliacaucana*, as a temporary mount **D** unencysted metacercaria isolated from *P.caucana*, as a permanent slide **E, F** paratype specimens, as temporary mounts **G** holotype adult specimen, isolated from *A.latifrons*, as a permanent slide. Scale bars: 20 μm (**A, B**); 20 μm (**C, D**); 100 µm (**E–G**).

We report a new species of *Oligogonotylus* from Colombia based on new morphological, molecular, and ecological evidence from a total of 1,306 worms. This taxon has a three-host life cycle, with the snail *A.colombiensis* as the first intermediate host, *P.caucana* as the second intermediate host, and *A.latifrons* as the definitive host (Fig. 2).

### ﻿Taxonomy

**Family Cryptogonimidae Ward, 1917**

#### Genus *Oligogonotylus* Watson, 1976

##### ﻿ Oligogonotylus andinus sp. nov.

Taxon classificationAnimaliaOpisthorchiidaCryptogonimidae

04DACE4F-FDD5-5197-87B3-4A496872ACA4

http://zoobank.org/265EF8A3-982C-4EEA-AA61-3EF1737E6B49

###### Diagnosis.

Adult with longitudinal row of three or four sucker-like gonotyls. Vitelline follicles extending between level of pharynx and anterior margin of ovary and occupying 25–38% of body length.

###### Cercaria description

**Figure 3.**
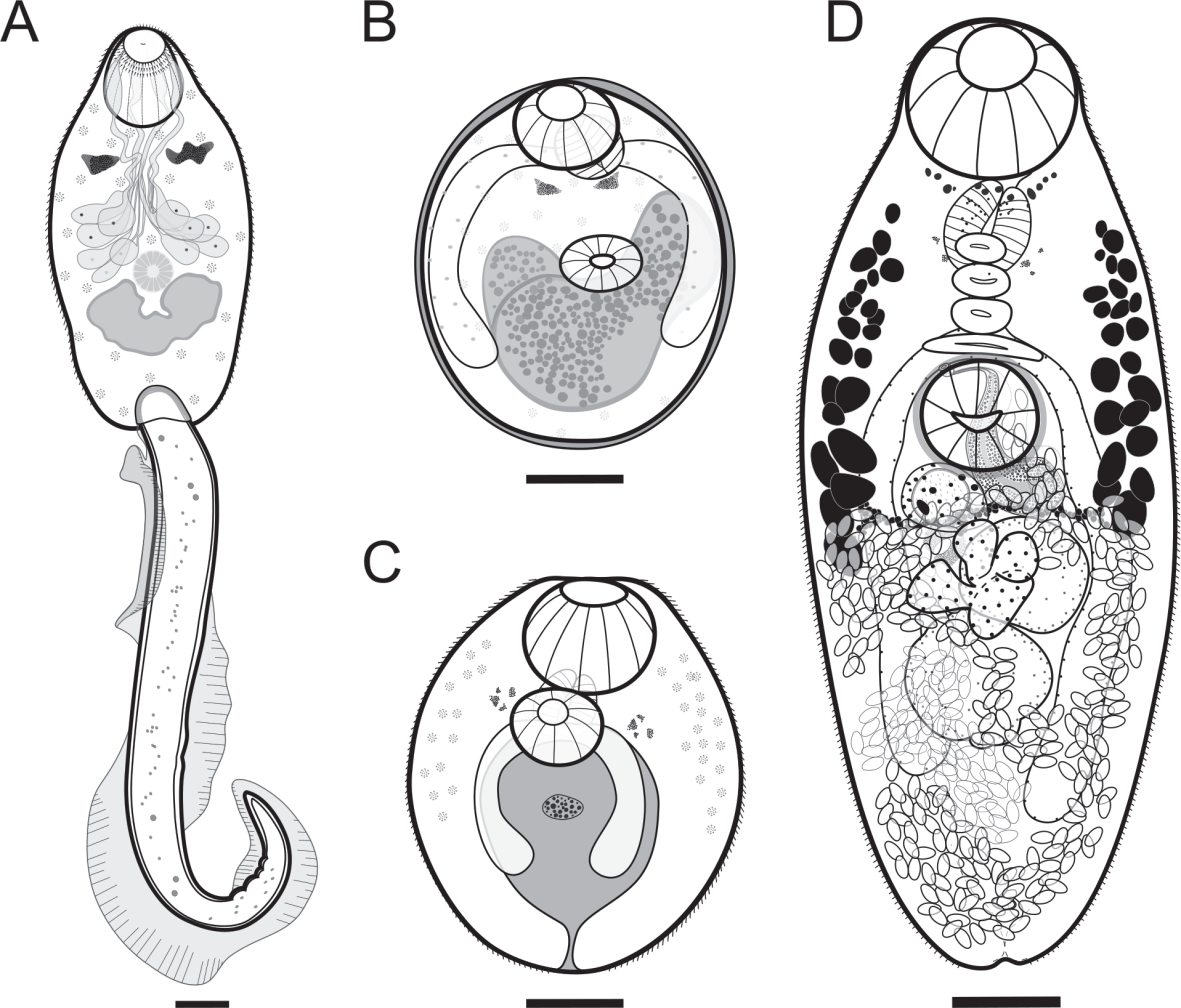
Developmental stages of *Oligogonotylusandinus* sp. nov. **A** pleurolophocercous cercaria emerged from *Aroapyrguscolombiensis***B** encysted metacercaria isolated from the muscle of *Poeciliacaucana***C** unencysted metacercaria isolated from *P.caucana***D** holotype, adult isolated from the large intestine of the trans-Andean cichlid *Andinoacaralatifrons*. Scale bars: 20 μm (**A**); 20 μm (**B, C**); 100 µm (**D**).

**(Figs 2A, 3A).** Pleurolophocercous type. Cercaria elongate, 149 (114–190) long by 82 (72–100) wide. Oral sucker 35 (18–46) long by 30 (24–34) wide, with three or four anterior rows of hook-like spines. Eyespots anterior to penetration glands. Pharynx located between eyespots. Esophagus and ceca undifferentiated. Penetration glands in middle body; seven pairs lead to exterior in four ducts running parallel to oral sucker in two bundles on each side. Rudimentary ventral sucker in middle body. Cystogenic glands on lateral margins of body. Excretory vesicle bilobed delimited anteriorly by primordial ventral sucker and posteriorly by insertion of tail. Tail longer than body with lateral fin, 294 (274–316) long by 22 (18–26) wide.

###### Metacercaria description

**(Figs 2B, 3B, C).** Encysted form spherical with thin cyst wall of variable thickness occupying entire cyst cavity (Fig. 3B). Cyst 281 (247–327) long by 193 (136–223) wide. Unencysted forms covered with numerous, simple, single-pointed tegumental spines which are smaller and less dense posteriorly. Oral sucker (Os) large, subterminal, 79 (58–98) long by 67 (50–84) wide. Ventral sucker (Vs) just pre-equatorial to equatorial, 53 (34–70) long by 49 (34–66) wide, smaller than oral sucker; Os/Vs (ratio) 1:0.7 (1:0.6–1:0.8) long by 1:0.7 (1:0.6–1:0.9) wide. Pharynx large, 43 (36–48) long by 38 (30–46) wide. Esophagus short; ceca wide, reaching middle hindbody. Remnants of eyespots at pharyngeal level. Genital primordium indistinct, posterior to ventral sucker. Excretory vesicle large, Y-shaped, with large lateral branches reaching anteriorly to level of ventral sucker and with short posterior duct opening at excretory pore.

###### Adult description

**Figure 4.**
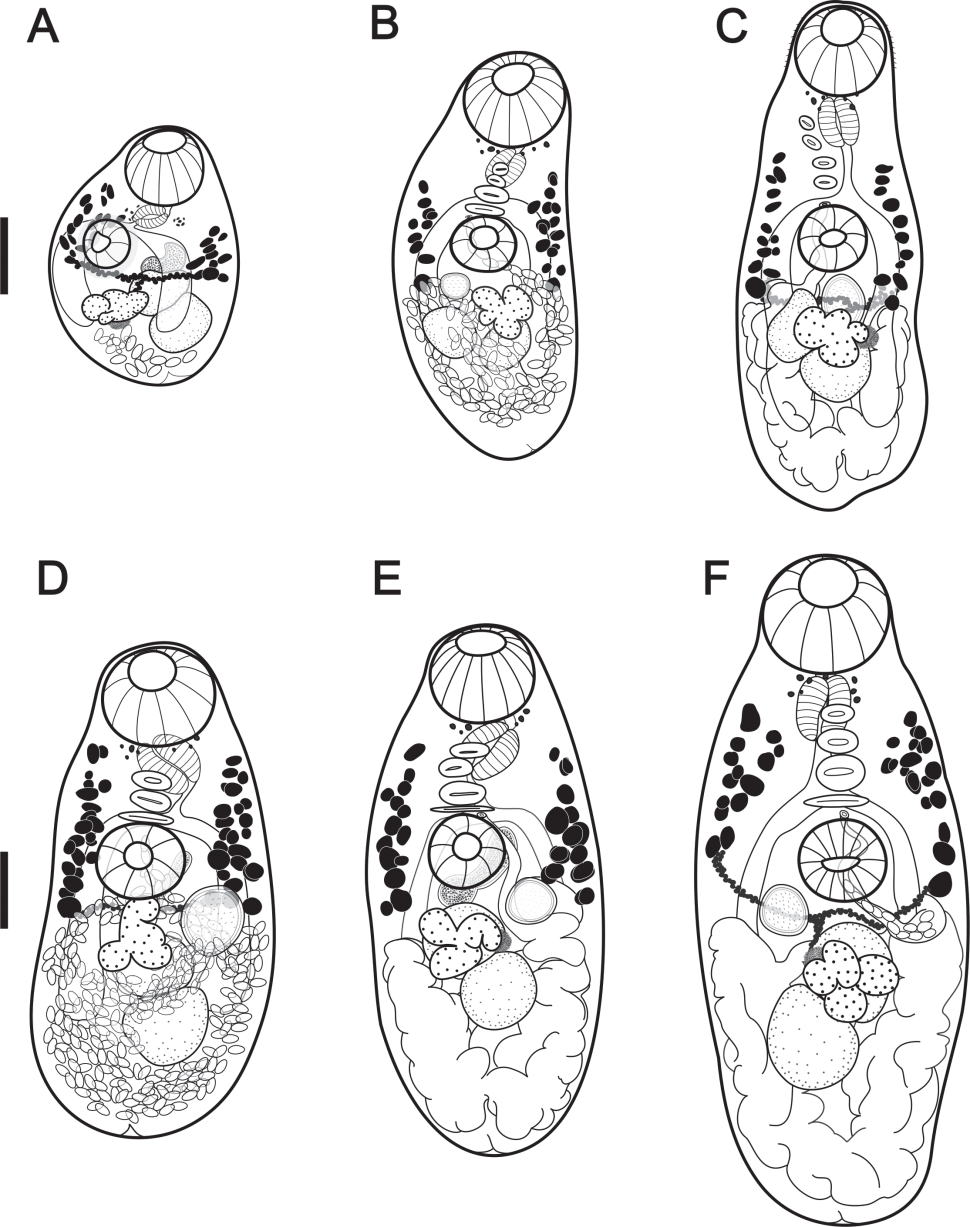
Paratypes of *Oligogonotylusandinus* sp. nov.; adults at different degrees of maturity **A** specimen in partly lateral orientation with the first eggs in utero; gonotyls were not observed **B, D–F** specimens with the seminal receptacle visible (left on **B** and **F**, right on **E** and **D**) and four gonotyls **A, C, D, F** specimens with the vitelline duct visible **A, D, E** specimens with the seminal vesicle dorsal to the ventral sucker. Scale bars: 100 μm (**A–F**).

**(Figs 3D, 4A–F).** Body oval to elongate-oval, widest at ovarian level, 517 (271–821, *n* = 21) long by 248 (167–335, *n* = 21) wide. Anterior and posterior ends of body rounded. Tegument spinose, with spines becoming shorter and less dense in posterior region. Remnants of eyespots diffuse and scattered at pharyngeal level. Oral sucker rounded, subterminal, without circumoral crown of spines, 106 (84–128, *n* = 21) long by 110 (66–144, *n* = 21) wide. Prepharynx short or inconspicuous, 18 (12–28, *n* = 9) long by 24 (16–28, *n* = 6) wide, with small extramural glands. Pharynx large, 59 (30–86, *n* = 18) long by 50 (26–70, *n* = 18) wide. Esophagus 40 (10–70, *n* = 13) long. Intestinal bifurcation immediately anterior to ventral sucker. Ceca reach just into post-testicular zone; distance from cecal extremities to posterior end of body 86 (60–114, *n* = 10). Longitudinal row of 4 (3–4, *n* = 17) sucker-like gonotyls located between mid-level of pharynx and anterior margin of ventral sucker, increasing in size from anterior to posterior. Ventral sucker rounded, pre-equatorial, 79 (56–100, *n* = 21) long by 78 (52–104, *n* = 21) wide, enclosed in ventrogenital sac, smaller than oral sucker; Os/Vs (ratio) 1:0.74 (1:0.61–1:0.88, *n* = 21) long by 1:0.72 (1:0.60–1:0.83, *n* = 21) wide; Os–Vs distance 112 (40–178, *n* = 15). Genital pore close to anterior margin of ventral sucker. Testes two, rounded to oval, in tandem, intercecal, in mid hindbody: anterior testis 72 (20–120, *n* = 20) long by 82 (50–112, *n* = 20) wide; posterior testis 80 (50–140, *n* = 21) long by 81 (46–140, *n* = 21) wide. Seminal vesicle tubulosaccular, undivided, mostly posterolateral to ventral sucker, 95 (30–128, *n* = 10) long by 82 (48–120, *n* = 11) wide. Cirrus sac and cirrus absent. Ovary lobed, median, post-equatorial, intercecal, between ventral sucker and posterior testis, partially overlaps anterior testis, 74 (38–108, *n* = 17) long by 90 (30–120, *n* = 17) wide. Seminal receptacle median, ovoid, dorsal, anterolateral to ovary, and ventral to vitelline duct, 65 (40–94, *n* = 11) long by 59 (40–82, *n* = 11) wide. Ootype posterolateral to ovary (on right side in type specimen and three paratypes, on left side in three other paratypes). Vitelline follicles small, irregular in shape to oval; arranged in lateral fields partly overlapping ceca or extracecal. Vitelline fields not confluent, extending from mid of pharynx to anterior region of ovary, occupying 32% (0.25–0.38, *n* = 15) of body length. Uterus in hindbody with several loops, passes medially between ovary and ventral sucker and forward to genital pore. Eggs small, 21 (18–24, *n* = 57) long by 10 (10–12, *n* = 57) wide. Excretory pore terminal. Excretory vesicle Y-shaped.

###### Taxonomic summary.

**Type specimens. *Holotype***: permanent slide; CCH.116: 170. ***Paratypes***: permanent slides; CCH.116: 171 to 178.

###### Type locality.

Colombia – Antioquia • Sopetrán, La Mirandita village; 6°30'42.0"N, 75°45'22.6"W; fishponds at 750 m a.s.l., February 2020, V. Vélez-Sampedro and C. Lenis leg.

###### Type hosts.

*Andinoacaralatifrons* (Steindachner, 1878) (Actinopterygii: Cichlidae).

###### Other hosts.

Cercariae in *Aroapyrguscolombiensis* Malek & Little, 1971 (Gastropoda, Cochliopidae); metacercariae in *Poeciliacaucana* (Steindachner, 1880) (Actinopterygii, Poeciliidae).

###### Prevalence

[**N (%)].** Cercariae 451 (2.0%); metacercariae 105 (38.1%); juveniles/adults 22 (100%).

###### Intensity

[**mean (range)].** Cercariae 8.6 (2–19); metacercariae 8.3 (5–14); juveniles/adults 41.1 (5–108).

###### Abundance

[**mean].** Cercariae (0.2); metacercariae (3.1); juveniles/adults (39.3).

###### Site in hosts.

Cercariae in hepatopancreas; encysted metacercariae in muscle; adults in medial region of large intestine.

###### Etymology.

The specific epithet (*andinus*) refers to the geographic distribution of the hosts, which are endemic to the Andes.

### ﻿Key to *Oligogonotylus* species

**Table:** 

1	Vitelline fields restricted to hindbody or barely extending forebody	**2**
–	Vitelline fields extended into forebody; between mid-region of pharynx and anterior margin of ovary; 3 or 4 gonotyls	***O.andinus* sp. nov.**
2	Vitelline fields between posterior margin of ventral sucker and posterior margin of posterior testis; 5–8 gonotyls	***O.manteri* Watson, 1976**
–	Vitelline fields between anterior margin of posterior testis and region of esophagus and pharynx; 6–8 gonotyls	***O.mayae* Razo-Mendivil et al., 2008**

### ﻿Molecular phylogenetic analyses

**Figure 5.**
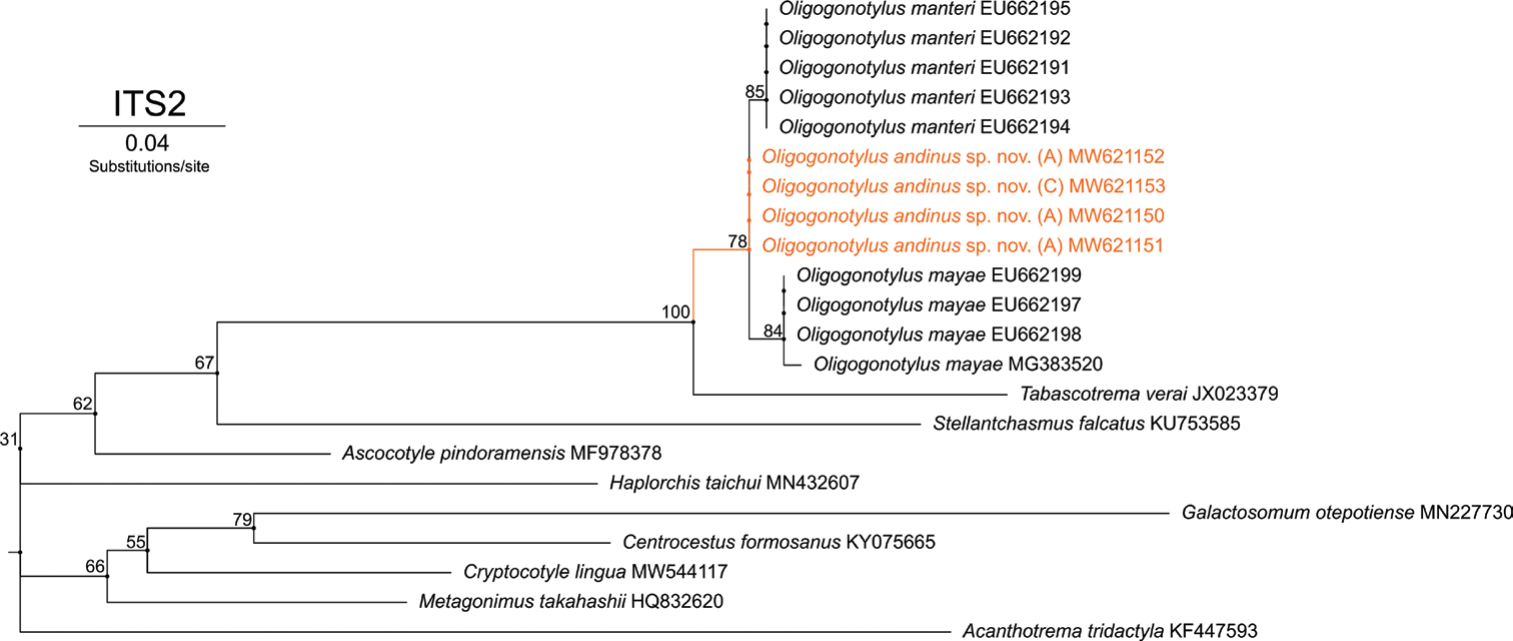
Phylogenetic relationships of ITS2 sequences of *Oligogonotylusandinus* sp. nov., *Oligogonotylusmanteri*, and *Oligogonotylusmayae*. Phylogenetic tree reconstructed using the maximum-likelihood method based on the symmetrical model with gamma distribution (SYM+G). Numbers on the branches correspond to the ultrafast bootstrap approximation support values. Each terminal is identified by the alphanumeric accession number of GenBank. Terminals in orange correspond to the sequences obtained in this report. Diagram made with IQ-TREE 2.1.3.

For the analysis of the ITS2 rDNA gene region, a total of four sequences from Colombia were obtained (GenBank MW621150, MW621151, MW621152, and MW621153). The final alignment includes 18 sequences from GenBank and consists of 22 sequences of 220 bp in total. The phylogenetic tree reconstructed by ML based on the SYM+G model (-lnL = 1232.8754) shows two clades within *Oligogonotylus* (Fig. 5). Clade І includes five sequences of O. *manteri* from Mexico, Belize, and Guatemala. Clade ІІ includes four sequences of O. *mayae* from Yucatán. Between these clades are the four sequences that represent the new species from Colombia forming a polytomy. The sequences of the cercaria (*n* = 1), metacercaria (*n* = 1, not included in the trees), and adults (*n* = 4, one not included in the trees) are identical, indicating that they belong to the same species. The bootstrap values are strong to include *O.andinus* sp. nov. in this genus and in the family Cryptogonimidae. The intraspecific genetic distances range from 0% (*O.andinus* sp. nov. and *O.manteri*) to 0.45% (*O.mayae*). The interspecific genetic distances (*p*-distances) are 0.45–0.46% for *O.andinus* sp. nov. versus *O.manteri*, 0.91–1.39% for *O.andinus* sp. nov. versus *O.mayae*, and 1.36–1.82% for *O.manteri* versus *O.mayae*.

**Figure 6.**
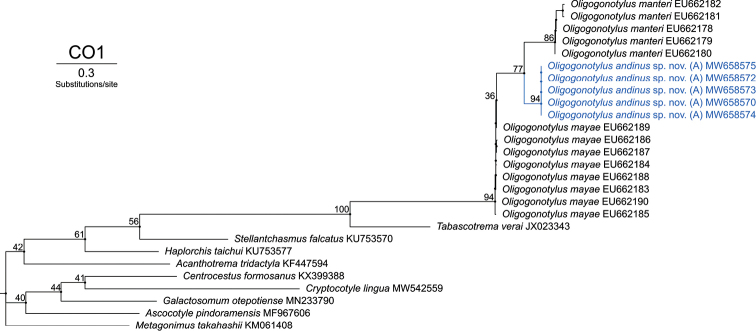
Phylogenetic relationships of COI sequences of *Oligogonotylusandinus* sp. nov., *Oligogonotylusmanteri*, and *Oligogonotylusmayae*. Phylogenetic tree reconstructed using the maximum-likelihood method based on the general time reversible with gamma distribution model (GTR+G). Numbers on the branches correspond to the ultrafast bootstrap approximation support values. Each terminal is identified by the alphanumeric accession number of GenBank. Terminals in blue correspond to the sequences obtained in this report. Diagram made with IQ-TREE 2.1.3.

For the analysis of the COI mtDNA gene region, five sequences from Colombia were obtained (GenBank MW658570, MW658572, MW658573, MW658574, and MW658575). The final alignment includes 22 sequences from GenBank and consists of 27 sequences of 330 bp in total. The phylogenetic tree reconstructed by ML based on the GTR+G model (-lnL = 2541.9707) shows two major monophyletic clades within *Oligogonotylus* (Fig. 6). Clade І includes five sequences of *O.manteri* from Mexico, Belize, and Guatemala. Clade ІІ includes five sequences representing the new species, all adult specimens. Clade ІІІ includes eight sequences of *O.mayae* from Yucatán that form a polytomy. The bootstrap values are strong (>85%) in each case supporting the position of the new species in *Oligogonotylus* and the family Cryptogonimidae. This topology identifies O. *andinus* sp. nov. and O. *manteri* as sister taxa with bootstrap support of 77%. The intraspecific genetic distances are 0% for *O.andinus* sp. nov., 0.28–3.66% for *O.manteri*, and 0.28–0.85% for *O.mayae*. The interspecific genetic distances (*p*-distances) are 10.27–11.28% for *O.andinus* sp. nov. versus *O.manteri*, 8.46–9.06% for *O.andinus* sp. nov. versus *O.mayae*, and 9.30–10.42% for *O.manteri* versus *O.mayae*.

**Figure 7.**
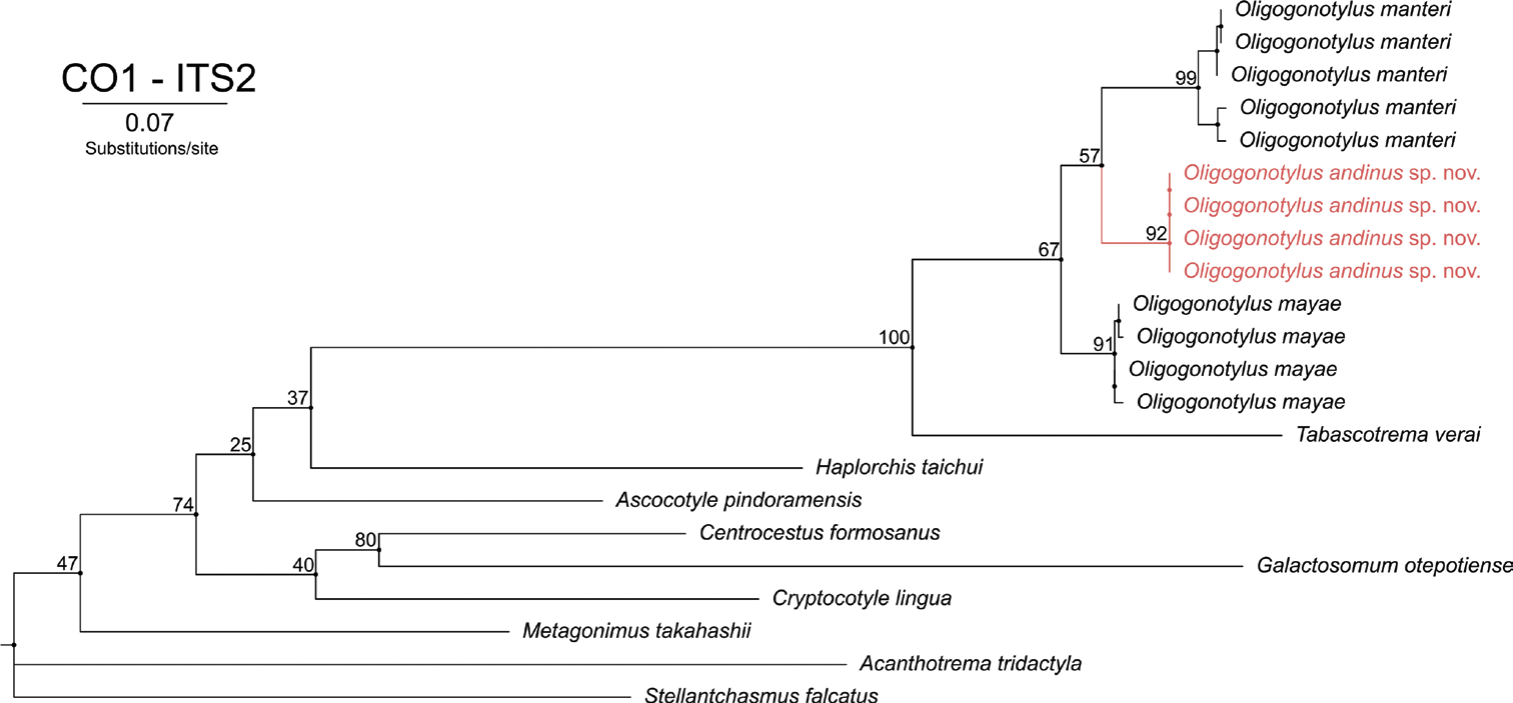
Phylogenetic relationships of COI and ITS2 concatenated sequences of *Oligogonotylusandinus* sp. nov., *Oligogonotylusmanteri*, and *Oligogonotylusmayae*. Phylogenetic tree reconstructed using the maximum-likelihood method based on the general time reversible with proportion of invariable sites and gamma distribution model (GTR+I+G) model. Numbers on the branches correspond to the ultrafast bootstrap approximation support values. Terminals in red correspond to the sequences obtained in this report. Diagram made with Garli IQ-TREE 2.1.3.

The extended analysis of concatenated COI and ITS2 sequences includes four sequences from Colombia. The final alignment includes 18 sequences from GenBank and consists of 22 sequences of 330 bp in total. The phylogenetic tree reconstructed by ML based on the GTR+I+G model (-lnL = 7111.7722) shows three major clades (Fig. 7) within *Oligogonotylus*. Clade І includes five sequences of *O.manteri* from Mexico, Belize, and Guatemala; clade ІІ includes the four sequences of *O.andinus* sp. nov.; and clade ІІІ includes four sequences of *O.mayae* from Yucatán. The three clades are monophyletic within a monophyletic genus, supporting the results of the COI topology for the new species and also giving more resolution to the phylogenetic relationships within the genus. The bootstrap values are strong (>90%) for clades I, II, and III, and this topology also identifies O. *andinus* sp. nov. and O. *manteri* as sister taxa. However, the bootstrap support is low (57%). The intraspecific genetic distances are 0% for O.andinus sp. nov., 0–2.18% for *O.manteri*, and 0.18–0.73% for *O.mayae*. The interspecific genetic distances (*p*-distances) are 6.36–6.96% for *O.andinus* sp. nov. versus *O.manteri*, 5.64–6.04% for *O.andinus* sp. nov. versus *O.mayae*, and 6.55–7.09% for *O.manteri* versus *O.mayae*.

The interspecific distances obtained from both the internal transcribed spacer (ITS2) and the mitochondrial gene (COI) together with the concatenated sequences provide clear support to the distinction of the new species. Our phylogenetic analysis using mitochondrial data shows that consistent with the wide distribution, the Middle American species have intraspecific mitochondrial variation —0.28–3.66% (*p*-distance)—over a wide latitudinal gradient from Costa Rica to Mexico. The intraspecific distance for the new species is null, consistent with all samples coming from a single Colombian population.

### ﻿Remarks

*Oligogonotylusandinus* sp. nov. represents the first species described in the Colombian cichlid *A.latifrons* and the third for the genus *Oligogonotylus*, with *O.manteri* and *O.mayae* as being parasites of Middle American cichlids. Morphological data from cercariae and metacercariae of *O.andinus* are compatible with those previously reported for *O.manteri* (see Scholz et al. 1994). The cercaria of the new species also corresponds to the pleurolophocercous type for having a lateral fin on the tail (Schell 1970; Yamaguti 1975; Scholz et al. 1994; Bray et al. 2008) and differs from that of *O.manteri* in having the tegument without hair-like sensory structures (Fig. 3A); hair-like sensory structures were reported on the body margins in *O.manteri*.

As adults, *O.andinus* sp. nov. is easily distinguishable from the other species in this group based on differences in the distribution of the vitelline fields as well as the number of gonotyls. The new species has vitelline fields between the mid-level of the pharynx and the anterior margin of the ovary and three or four gonotyls. *Oligogonotylusmanteri* has vitelline fields between the posterior margin of the ventral sucker and the posterior-end of the posterior testis and has five to eight gonotyls (Watson 1976), whereas *O.mayae* has vitelline fields from a level between the pharynx and the esophagus and the anterior margin of the posterior testis and six to eight gonotyls (Razo-Mendivil et al. 2008).

The analysis of the morphology of the reproductive system of *O.andinus* sp. nov. at different stages of maturity (Figs 3D, 4A–F) reveals that the seminal receptacle is ventral to the vitelline duct; the ootype complex overlaps the lateral region of the ovary dorsally (on right side in the type specimen and three paratypes but on left side in three other paratypes, on the same side of the seminal receptacle); and the distal region of the uterus and the seminal vesicle reach the anterior margin of the ventral sucker and converge at the genital pore. These features are not detailed in the description of *O.manteri*. In *O.mayae*, as in the new species, the seminal vesicle is tubulo-saccular and posterolateral to the ventral sucker.

Morphology-based taxonomic conclusions are supported by the analysis of molecular data from the ITS2, COI and COI–ITS2 barcoding. Our analyses show that the specimens from Colombia represent a new species. The polytomy formed by the sequences of the new species in the phylogenetic tree of ITS2 is due to the low rate of mutation that this molecular marker has. In conjunction, the phylogenetic trees of COI and the concatenated sequences of COI and ITS2 show that all isolates from Sopetrán represent a reciprocally monophyletic assemblage, distinct from isolates of *O.manteri* and *O.mayae*, which are shown in both topologies to be sister taxa.

## ﻿Discussion

In the present study, *O.andinus* sp. nov. is described and associated with three endemic Andean hosts from Sopetrán, Antioquia, Colombia. Initially, molecular analyses of cercariae which had emerged from *A.colombiensis* enabled identification to the generic level. Subsequently, the location of the focus in the fishponds facilitated the life cycle study using five species of fishes present in the fish farm; two are endemic species (*P.caucana* and *A.latifrons*), and three are exotic cultured species (*O.aureus*, *C.macropomum*, and *O.mossambicus*). Of these, the two endemic fishes act as intermediate and definitive hosts of the parasite, respectively. The high prevalence of *O.andinus* in *P.caucana* (38.1%) and *A.latifrons* (100%) are a result of at least three factors: (a) the confinement of the snails and fishes in small semi-closed ponds, which increases the rate of parasite-host encounters; (b) the establishment of a large population of *P.caucana* in the fishpond, with few predators (*A.latifrons* and birds such as kingfishers); and (c) a voracious diet of *A.latifrons* that included snails, poecilids, and insects (judging from the stomach content; present study). Despite their close confinement, the farmed fish were negative for the new species because their diets are based on plants and fish feed.

Previous studies have shown that both *O.manteri* and *O.mayae* use the cochliopid snail *P.coronatus* (misidentified as *Benthonellagaza*), and that *O.manteri* also uses the cochliopid snail *Aroapyrgusalleei* Morrison, 1946 as the first intermediate host and the cichlid fish *M.urophthalmus* either as the second intermediate or the main definitive host among other 11 species of cichlids (Scholz et al. 1994; Jiménez-García and Vidal-Martínez 2005; Pérez-Ponce de León et al. 2007; May-Tec et al. 2020; Velázquez-Urrieta and Pérez-Ponce de León 2021). *Oligogonotylusandinus* sp. nov. is related to hosts whose history has been shaped by the Andes Mountains and whose life cycle occurs in lentic systems. A cochliopid snail (*A.colombiensis*) is the first intermediate host, a poecilid fish (*P.caucana*) is the second intermediate host, and a trans-Andean cichlid fish (*A.latifrons*) is the definitive host; thus, the present work extends the *Oligogonotylus*–Cochliopidae–Cichlidae association. Therefore, species of *Oligogonotylus* are still considered as members of the core helminth fauna of cichlids (Pérez-Ponce De León and Choudhury 2005), but also as members of the core helminth fauna of cochliopids (May-Tec et al. 2020; Velázquez-Urrieta and Pérez-Ponce de León 2021; present study).

*Aroapyrguscolombiensis* is distributed in the Magdalena–Cauca basin and inhabits small lotic systems with abundant stretches of slow water and decomposing dead leaves (Linares et al. 2018). It has been reported as a host for *Paragonimuscaliensis* Little, 1968 in Valle del Cauca (Malek and Little 1971) and Antioquia (Lenis et al. 2018). *Poeciliacaucana*, which is commonly called Pipón, Piponcita, or Cauca Molly, is distributed in the hydrographic slopes of the Pacific and the Caribbean, especially in the Magdalena–Cauca basin (Castellanos-Morales et al. 2011; Martínez et al. 2016; Mojica et al. 2018; Ruiz-Guerra and Echeverry-Galvis 2019). Normally it inhabits rivers and brooks (Roman-Valencia 1990), but small streams also lead it to lagoons and natural or artificial ponds where it is reported in Magangué, Bolívar, Los Palmitos, Sucre (Navarro et al. 2019), and Sopetrán (present study). *Andinoacaralatifrons* is colloquially named Blue mojarra and it is grouped in two lineages in Colombia, (1) the upper Magdalena and Catatumbo clade, and (2) the upper Cauca and upper Magdalena clade (De la Ossa-Guerra et al. 2020), where our host population belongs. It inhabits muddy waters, clean-water streams, and lentic environments. It feeds on worms, insects, plant materials, fish remains, debris, and other live prey, such as crustaceans (Maldonado-Ocampo et al. 2006; Olaya-Nieto et al. 2016), making it susceptible to infection by trematodes.

This is the first study of the helminth fauna associated with *A.colombiensis* and *A.latifrons* and extends the known geographic distribution of *Oligogonotylus* spp. from Middle America to South America (Choudhury et al. 2016, 2017) where *O.andinus* sp. nov. was found at 750 m above sea level. Further field-based studies must include an increased sampling size to determine whether major intraspecific distances exist or if there are other populations in the Cauca river basin, and to reveal the ecogeographic limits of *Oligogonotylus* spp. in Colombia, particularly in areas with a likely suitable habitat where the species has yet to be found. Future taxonomic and ecological host-parasite research must lead to an understanding of fish parasite diversity and the risk of the transmission of zoonotic diseases, considering the dependence on fish as a food resource in rural areas of Colombia.

## Supplementary Material

XML Treatment for Oligogonotylus andinus
